# Microbial Flora Changes in Cesarean Section Uterus and Its Possible Correlation With Inflammation

**DOI:** 10.3389/fmed.2021.651938

**Published:** 2021-11-22

**Authors:** Xing Yang, Xinyi Pan, Meihong Cai, Bolun Zhang, Xiaoyan Liang, Guihua Liu

**Affiliations:** ^1^Reproductive Medicine Centre, The Sixth Affiliated Hospital of Sun Yat-sen University, Guangzhou, China; ^2^Reproductive Medicine Centre, Guangzhou First People's Hospital, School of Medicine, South China University of Technology, Guangzhou, China

**Keywords:** microbial flora, microbial diversity, cesarean section, inflammation, cytokines

## Abstract

**Background:** It has not been fully elucidated whether the change of the uterus flora is correlated to impaired fecundity. This case-control study aimed to analyze the differences in uterus microbial flora between women with post-cesarean section (CS) scar diverticulum (PCSD) (CS group) and women after vaginal delivery (control group), exploring the correlation between differentially expressed microbial flora and inflammation.

**Methods:** Infertile women who underwent hysteroscopy were enrolled in this case-control study. The swab samples were classified into four subgroups: CS cervix group, CS endometrium group, control cervix group, and control endometrium group. The total DNA obtained from 16 women (a total of 31 samples, the cervix or endometrium) was extracted for 16S recombinant DNA (rDNA) analysis. The Luminex platform was used to detect the abundance of 34 kinds of local inflammatory cytokines in 32 endometrium samples, and the correlation between microbial flora and inflammatory cytokines was analyzed.

**Results:** The alpha and beta diversity analysis indicated that the microbial diversity was higher in the CS group compared to the control group, especially in endometrium tissues. The heatmaps revealed that the microbial flora structure differs at each level of the phylum-class-order-family-genus among the groups. The analysis of four of the most prominently changed microbial flora revealed that *Lactobacillus* in the cervix was significantly higher in the control group when compared with the cesarean section group (*P* < 0.05). Furthermore, *Proteobacteria* and *Neisseriaceae* had a higher abundance in the CS groups, especially in the cervical tissue (*P* < 0.05), while *Staphylococcaceae* increased only in the CS endometrium tissue (*P* < 0.05). Next, these women were re-divided into the high- and low-*Staphylococcaceae*, and the abundance of 34 kinds of local inflammation cytokines was compared between groups. It was found that there was a positive correlation between *Staphylococcaceae* and IL-2, and a negative correlation between *Staphylococcaceae* and IL-8 (*P* < 0.05).

**Conclusion:** The present results suggest that the disrupted uterus microbiota composition in women with CS may be closely associated with local inflammation. The interplay between the microbiota and the immune system may be linked to clinical disorders. The potential mechanisms require further exploration.

## Introduction

Cesarean section operation can significantly reduce the dystocia and stillbirth rate. However, cesarean section (CS) rates are increasing globally. In China, the CS rate has increased from 28.8% in 2008 to 36.7% in 2018, especially for women who received assisted reproduction technology (ART) treatment (1). Researchers have reported a lower pregnancy rate in patients with previous CS delivery when compared with patients with previous vaginal delivery after ART (2), and the potential causes remain obscure. The poor healing of local incisions after CS possibly forms a local nick, and connects with the uterine cavity, leading to post-CS scar diverticulum (PCSD). Due to the long-term poor drainage of menstrual blood, PCSD patients are often accompanied with uterine cavity effusion, which manifests as menostaxis (3), and this may further cause chronic endometritis (CE), interrupted endometrial receptivity, and impaired fecundity (4).

Although the endometrium has not been extensively studied as a site of commensal bacterial colonization, it was reported that the endometrium harbors a significantly lower quantity of microbes, which are dominated by Lactobacilli, Bifidobacteriaceae, and Streptococcaceae (5). Diverse dominant flora among populations with different pathological characteristics have been reported. For example, Moreno et al. reported that the presence of a non-lactobacillus-dominated microbiota (<90% *Lactobacillus*, containing over 10% of other bacteria) in a receptive endometrium was associated with significant decreases in implantation rate (6). The low detection rate of lactobacilli in women with CE also suggests that the changes in the endometrium flora may be correlated to secondary infertility (7). Microbial flora disruption can be a vital component for CE and impaired fecundity.

It is well-known that immune adaptation is pivotal in regulating the establishment and maintenance of pregnancy and the implantation phase is characterized by low-grade proinflammatory immune reactivity, including the production of major cytokines IL-6, IL-8, and tumor necrosis factor (TNF)-a. This response was considered to support the local repair of endometrial injury and the removal of cellular debris during trophoblast invasion and implantation (8). The implantation procedure is directly affected by local microorganisms (9). The interaction between the microbiota and the immune system is a complex process, which is crucial for maintaining normal homeostasis in organs, albeit under the influence of several constitutional and environmental factors. The immune system surveys the microbiota and detects alterations through tonic sensation via immune pattern recognition receptors, which may have been proved to explain the mechanism of some autoinflammatory disorders, such as asthma (10).

However, the detailed mechanisms of the uterine incision diverticulum that result in a decreased implantation rate have not been fully elucidated. The present study aimed to analyze the potential relationship between microbial flora and the inflammation in the reproductive tract between subfertile CS and control women. Paired cervix and endometrium microflora were analyzed by 16S recombinant DNA (rDNA) sequencing. A total of 34 kinds of cytokines and chemokines targeted by T-cells and macrophages were detected since these are the critical components of the innate immune system in the epithelial of the uterus. The correlation between different microbial flora and inflammatory factors was further explored, to initially provide clues for the correlation between the microbial flora, inflammation, and infertility.

## Methods

### Patient Recruitment

Women with PCSD (CS Group) or those who had natural vaginal deliveries (control group) were recruited from the Sixth Affiliated Hospital of Sun Yat-sen University in 2019. All the study procedures were reviewed and approved by the ethics review board of the hospital (IRB no. 2019ZSLYEC-005S). The inclusion criteria were as follows: (1) within 18–45 years old; (2) secondary infertility with previous CS or natural vaginal delivery; (3) informed consent. The exclusion criteria were as follows: (1) unilateral oophorectomy, hysteromyomectomy, or adenomyomectomy; (2) uterine abnormalities: malformation, submucosal fibroids, or uterine adhesions; (3) endometriosis or adenomyosis; (4) recurrent miscarriage; (5) acute pelvic inflammation, cervicitis, or vaginitis; (6) history of tuberculosis infection; (7) immunological disease; (8) received therapeutic medicine that may affect the survival status of bacteria in the past month, such as antibiotics, glucocorticoids, or immunosuppressants.

### Swab Sample Preprocessing

During the speculum exam when the patients were undergoing a hysteroscopy, a single-tipped CultureSwab was inserted into the endocervix to the depth of the entire tip of the swab, rotated 360°, held for 10 s, and removed, avoiding contact with the vaginal walls. Then, a bilayer assembling tube was inserted into the cervix, and the first inner swab was inserted further into the uterus and rotated 360°, held for 10 s, and removed. The swabs used to detect the microbial flora were immersed in 500 μl of a DNA isolation buffer. A second inner swab was used for sampling in the same manner to detect cytokine profiling. This was immersed in 500 μl of 15-mM Tris, pH 7, and 2 mM of ethylenediaminetetraacetic acid. The swabs were preprocessed and stored at −80°C within the hour after collection. Then, the samples were divided into four groups for the microbial flora detection: CS cervix group, CS endometrium group, control cervix group, and control endometrium group.

### 16S rDNA Sequencing

The total DNA obtained from the cervical and endometrial tissues were extracted using a DP328 DNA extraction kit (Tiangen, Beijing, China), according to the manufacturer's protocol. The DNA was confirmed with 1% agarose gel electrophoresis, and the concentration was detected using a dsDNA HS kit (Qubit, Thermo Fisher, Waltham, Massachusetts, United States). The amplification and sequencing were performed on the Illumina HiSeq2500 (Illumina, San Diego, California, United States) platform using the pair-end (PE) method of end sequencing.

### 16S rDNA Analysis

The original PE reads were cut, and the low-quality reads were filtered out by SOAPnuke [BGI-flex Lab (China), website: https://github.com/BGI-flexlab/SOAPnuke] with default parameters to obtain the effective data clean reads. Then, these clean reads were further spliced into Tags through the overlap, and the target Tags fragments were obtained. Afterward, these Tags fragments were clustered into Operational Taxonomic Units (OTU) using QIIME [Rob Knight Lab (USA), website: http://qiime.org/] and annotated to obtain the microbial information in a community. The extracted sequence information in the OTU was according to the phylum, class, order, family, and genus, respectively. The relative abundance of each species was calculated, and the species with a relative abundance above 1% were selected to draw the relative abundance of each species. An abundance distribution map diversity analysis, including alpha diversity (Observed_species index, Chao1 index, Shannon index, and Simpson index), was performed by QIIME to evaluate the species richness and uniformity, and beta diversity was adopted to calculate the distance between groups with a Wilcoxon rank-sum test. A Venn diagram was used to analyze the common and unique OTU information among the different groups. The phylogenetic tree was constructed based on the OTU representative sequence. The community structure differences were further compared using Principal Co-ordinates Analysis (PCoA) based on the weighted unifrac distance and the non-metric multidimensional scaling (NMDS) analysis for samples in multidimensional space were simplified to low-dimensional space for positioning, analysis, and classification. The linear discriminant analysis effect size (LEfSe) software [The Huttenhower Lab (USA), website: https://huttenhower.sph.harvard.edu/lefse/] used a linear discriminant analysis (LDA) statistical model in which the interclass dispersion matrix of the projected pattern samples can be maximized and the intraclass dispersion matrix can be minimized, to verify the differences in the composition and community structure of the species. The LEfSe used the phylum, class, order, family, and genus-level abundance table as input, based on the relationship tree of the species to calculate the LDA score of each level between two groups.

### Cytokines Detection

The intrauterine swab sample was eluted and detected for cytokines using the Luminex multiplex cytokine panel (Bio-Rad, Hercules, California, United States) in a T-cell and macrophage-targeted multiplex panel according to the instructions of the manufacturer. The 34 kinds of cytokines and chemokines were the MIP-1alpha, SDF-1alpha, IL-27, IL-1beta, IL-2, IL-4, IL-5, IP-10, IL-6, IL-7, IL-8, IL-10, Eotaxin, IL-12p70, IL-13, IL-17A, IL-31, IL-1RA, RANTES, IFN-γ, GM-CSF, TNF-α, MIP-1β, IFN-α, MCP-1, IL-9, TNF-β, GRO-α, IL-1α, IL-23, IL-15, IL-18, IL-21, and IL-22. The content of each cytokine in the samples was quantified according to the standard curve established by the standard substance. The reference values were derived from normal serum samples inside the laboratory.

### Statistical Analysis

The quantitative data were expressed as mean ± SD. A Wilcoxon rank-sum test was used to compare the microbial or cytokines abundance among different tissues in the CS group and control group as all data are skewed data. The patients were further divided into the high and low *Staphylococcacea* group, based on the endometrial *Staphylococcacea* median abundance (the cutoff level was 2%), to compare the cytokines abundance difference. All statistical analyses were performed using SPSS v22.0 (SPSS Inc., Chicago, USA). *P* < 0.05 indicated that the difference was statistically significant.

## Results

### Microbial Flora Diversity Analysis

**Figure 1:**
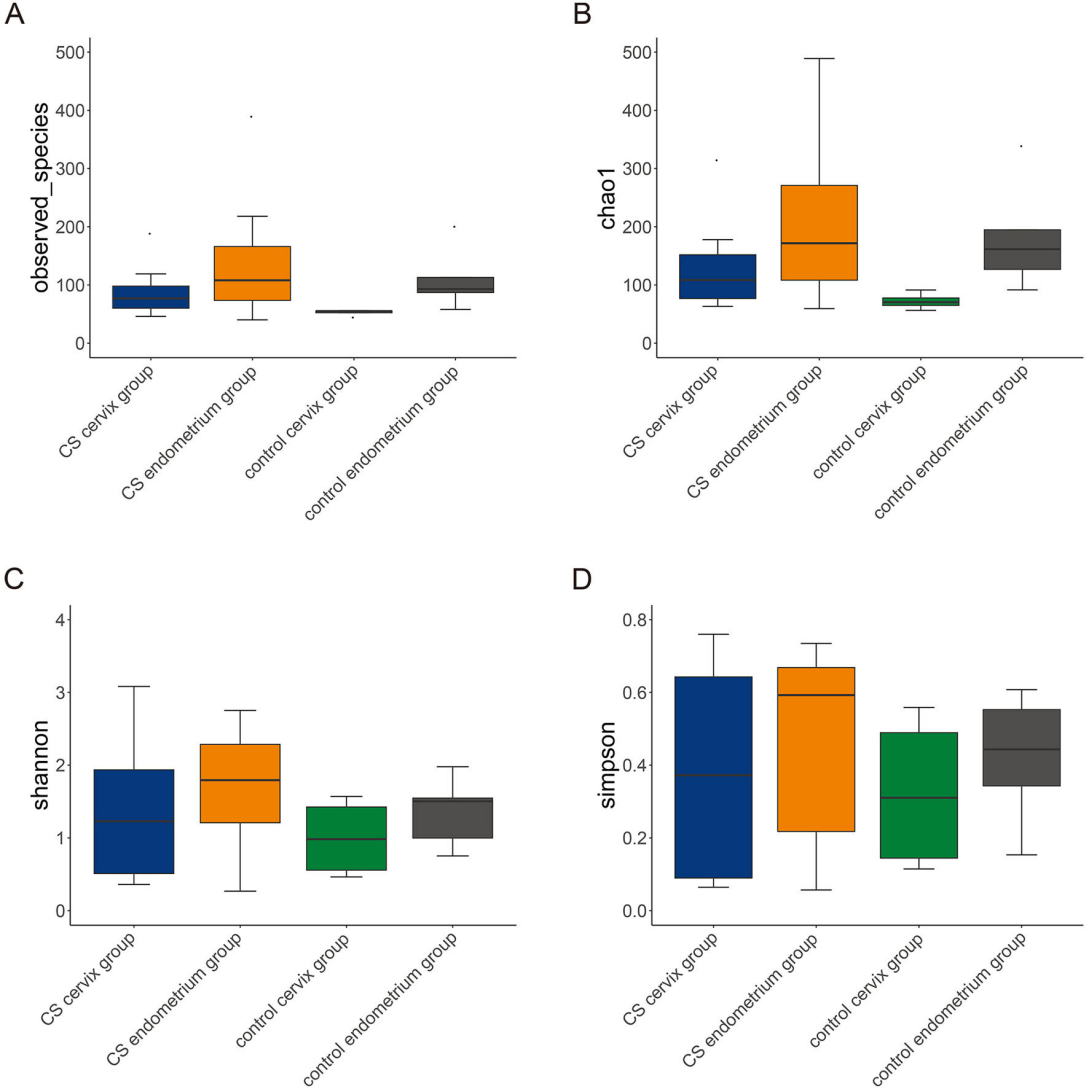
Alpha diversity. The alpha diversity, including observed species index **(A)**, Chao index **(B)**, Shannon index **(C)**, and Simpson's diversity index **(D)**. The cesarean section group had a higher number of microbial species and greater diversity, when compared to the control group. In particular, there was a higher diversity index in the endometrium, when compared to cervix tissues, in each group.

**Figure 2:**
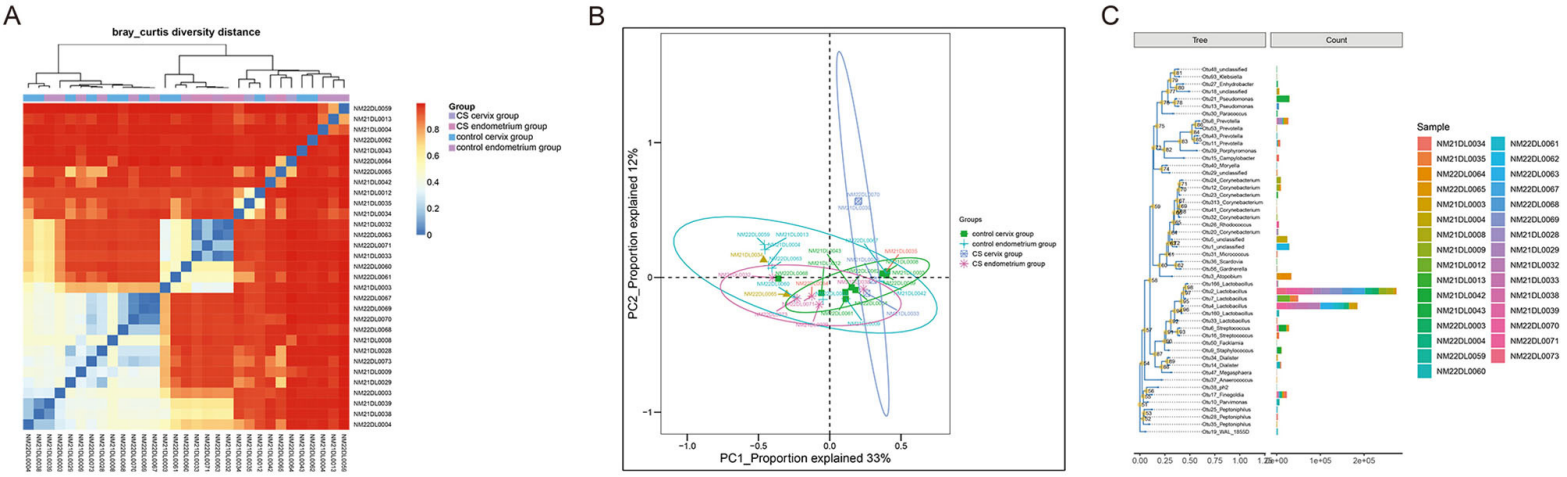
Beta diversity analysis. **(A)** Distance heatmap; **(B)** PCoA analysis; **(C)** phylogenetic tree with the top 50 OTUs, suggesting that *Lactobacillus* is the dominant bacteria in all samples.

A total of 16 women were included in the present study, which included nine women in the CS group and seven women in the control group. The women in the CS group were 32.26 ± 3.56 years old and were 33.86 ± 3.13 years old in the control group. The paired cervix and endometrium tissues samples were, respectively, collected. The total number of effective sequences in the four groups was 10,586,943. Alpha diversity was used to analyze the richness and uniformity of the species composition. Among these, the observed species index, Chao index, Shannon index, and Simpson's diversity indicated that the CS group had a higher number of microbial species and a greater diversity when compared to the control group. In particular, the endometrium tissues had a higher diversity index score than the cervix tissues for each individual (Figure 1). Next, beta diversity was used to calculate the distance among groups, and evaluate the biological diversity. The significant difference in distance between the cervical and endometrial tissues was presented through the heatmap and PCoA analysis (Figures 2A,B). The top 50 OTUs with overall relative abundances were selected to construct a phylogenetic tree. This suggested that Lactobacillus is the dominant bacteria in both the endometrium and cervix samples (Figure 2C).

### Clustering Analysis

**Figure 3:**
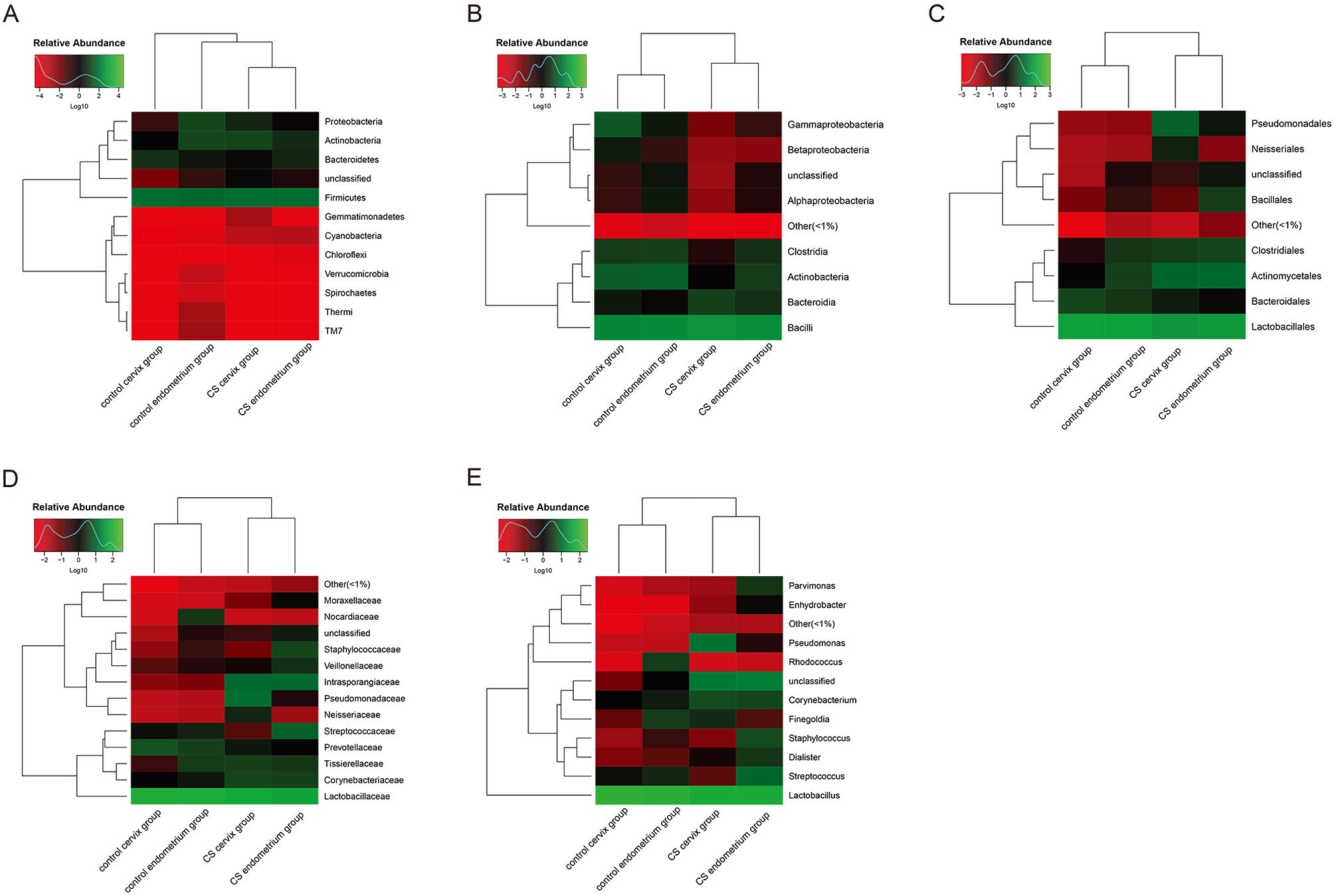
Heatmaps for the clustering analysis. **(A)** Heatmap at the phylum level; **(B)** heatmap at the class level; **(C)** heatmap at the order level; **(D)** heatmap at the family level; **(E)** heatmap at the genus level. The color scale bar ranged from −2.0 to 2.0, with green, black, and red representing the low (green), medium (black), and high (red) microbial abundance, respectively.

Heatmaps were used to assess the composition and abundance of microbial flora among the groups at the phylum-class-order-family-genus level. The species with a relative abundance of ≥1% were used for the heatmap analysis. The heatmaps at the level of the phylum (Figure 3A), class (Figure 3B), order (Figure 3C), family (Figure 3D), and genus (Figure 3E) indicate that the paired endometrium and cervix sample obtained from the same individual is similar in microbial flora structure at each level (Individual information in Supplementary Figure).

### Dominant Microbial Flora at Different Levels

**Figure 4:**
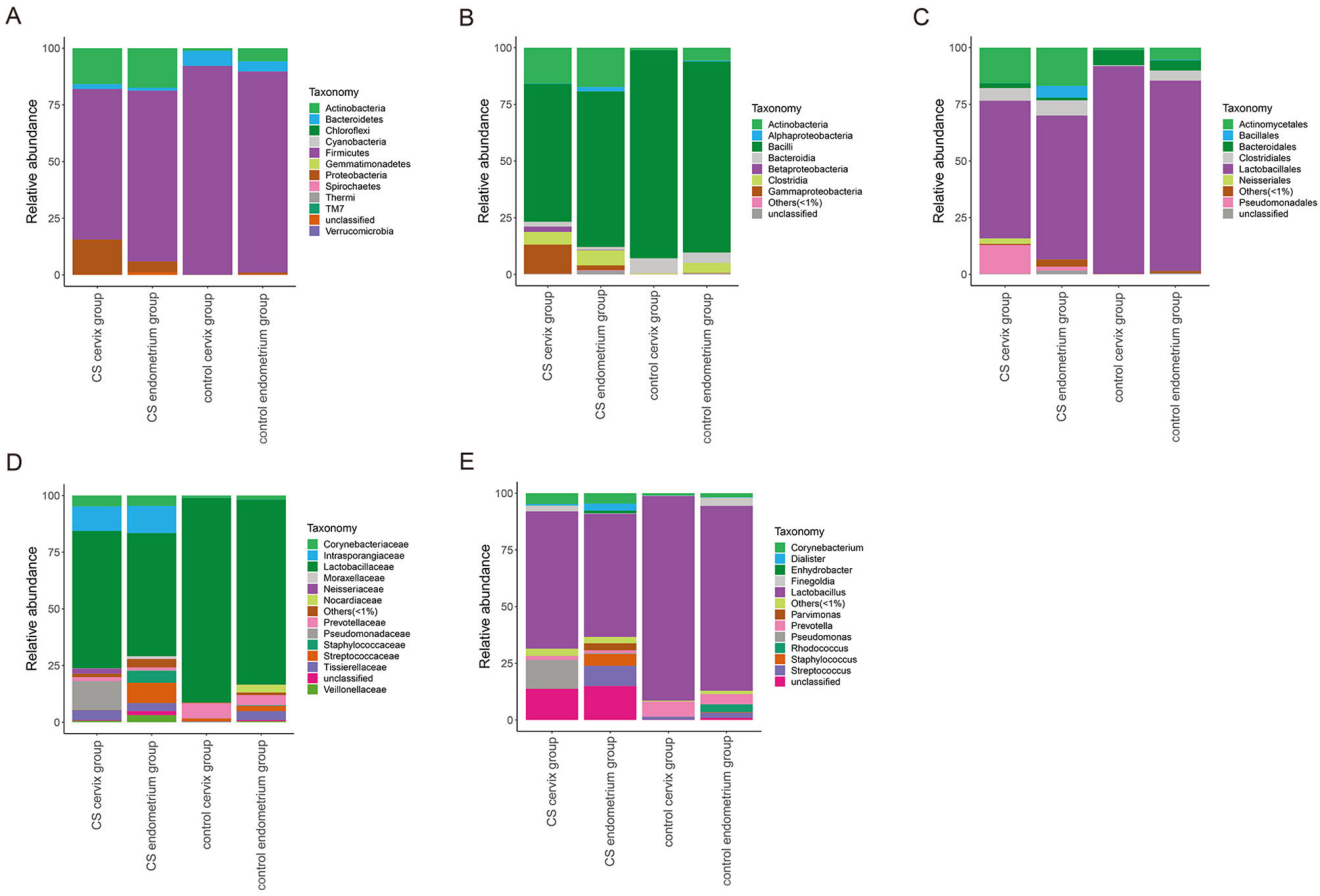
The relative abundance of microbial flora at the phylum-class-order-family-genus level. The relative abundance of species at the phylum **(A)**, class **(B)**, order **(C)**, family **(D)**, and genus **(E)** levels. The figure shows the species with a relative abundance of ≥1%. The abundance was lower than 1%, and those classified as unclassified or unidentified were classified as others.

The relative abundance of species was analyzed using the OTUs. The species with a relative abundance ≥1% were used for the bacteria analysis. (1) A total of 12 microbial flora phyla were found in each group. In the control groups, the dominant abundance of bacteria within the phylum was *Firmicutes* (cervix 92.13%, range 71.52–99.70% and endometrium 88.70%, range 70.85–99.47%), and the abundance decreased in the CS cervix group (66.40%, range 1.87–99.53%) and CS endometrium group (75.36%, range 1.54–99.67%). The second dominant abundance within the phylum was *Actinobacteria* (endometrium 1.13%, range 0.14–3.94% and cervix 5.77%, range 0.15–17.32%) in the control group. This increased in the CS cervix group (15.84%, range 0.16–97.30%) and CS endometrium group (17.40%, range 0.09–84.21%) (Figure 4A). (2) A total of nine microbial flora classes were found in each group. In the control group, the dominant abundance within the class was *Bacilli* (endometrium 91.75%, range 71.40–99.47% and cervix 84.27% range 60.39–95.44%), and this decreased in the CS cervix group (60.82%, range 0.91–98.60%) and CS endometrium group (68.65%, range 0.89–99.41%). The second dominant abundance within the class was *Actinobacteria* (endometrium 1.09%, range 0.10–3.90% and cervix 5.73%, range 0.11–17.29%) in the control group. This increased in the CS cervix group (15.76%, range 0.13–97.17%) and CS endometrium group (17.33%, range 0.07–84.04%) (Figure 4B). (3) A total of nine microbial flora orders were found. In the control group, the dominant abundance within the order was *Lactobacillales* (endometrium 96.71%, range 91.38–99.42% and cervix 83.98%, range 60.35–95.38%), and this significantly (Wilcoxon rank-sum test, *P* = 0.017) decreased in the CS cervix group (60.74%, range 0.80–98.51%) and the CS endometrium group (63.47%, range 0.81–99.38%). The second dominant abundance within the order was *Actinomycetales* (endometrium 1.06%, range 0.10–3.90% and cervix 5.32%, range 0.08–17.29%) in the control group, and this increased in the CS cervix group (15.65%, range 0.09–97.16%) and CS endometrium group (16.88%, range 0.07–84.02%) (Figure 4C). (4) A total of 14 microbial flora families were found. In the control group, the dominant abundance within the family was *Lactobacillaceae* (endometrium 95.30%, range 91.26–99.19% and cervix 81.56%, range 60.20–95.19%), and this significantly (Wilcoxon rank-sum test, *P* = 0.038) decreased in the CS cervix group (60.58%, range 0.70–98.27%) and CS endometrium group (54.31%, range 0.73–99.23%). The second dominant abundance within the family was *Prevotellaceae* (endometrium 6.63%, range 0.01–24.43% and cervix 4.49%, range 0.01–18.97%) in the control group, and this decreased in the CS cervix group (1.69%, range 0.01–11.50%) and CS endometrium group (1.10%, range 0.01–6.14%) (Figure 4D). (5) A total of 13 microbial flora genera were found. In the control group, the dominant abundance within the genus was *Lactobacillus* (endometrium 95.30%, range 91.26–99.19% and cervix 81.56%, range 60.20–95.19%). *Lactobacillus* significantly (Wilcoxon rank-sum test, *P* = 0.038) decreased in the CS cervix group (60.58%, range 0.70–98.36%) and CS endometrium group (54.31%, range 0.73–99.23%). The second dominant abundance within the genus was *Prevotella* (endometrium 6.65%, range 0.01–24.43% and cervix 4.49%, range 0.01–18.97%) in the control group, and this decreased in the CS cervix group (1.69%, range 0.01–11.50%) and CS endometrium group (1.10%, range 0.01–6.14%) (Figure 4E).

### Characteristics of the Flora in the CS Group

**Figure 5:**
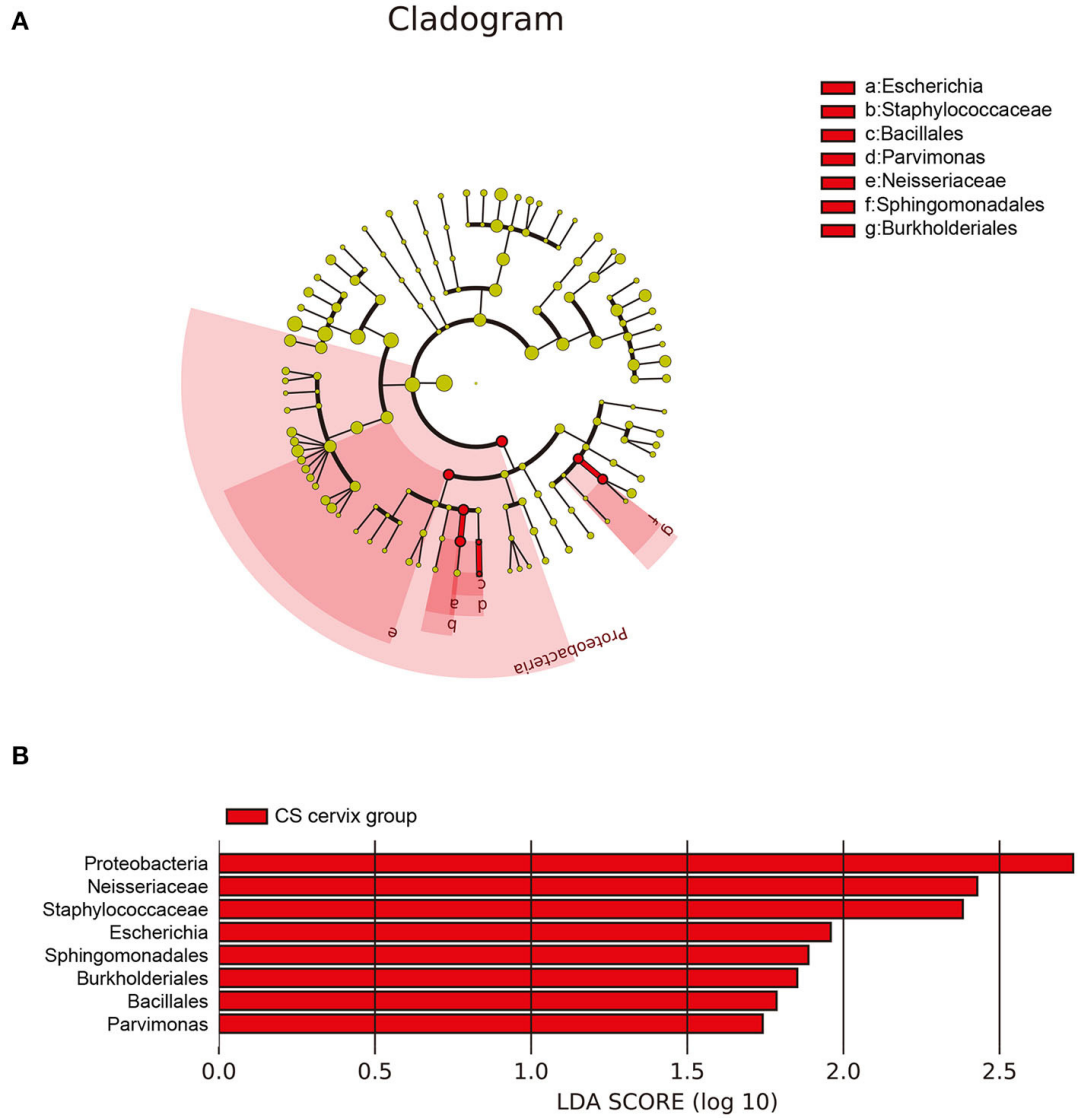
The differences in abundance of bacteria genus among groups. **(A)** The LEfSe clustering tree: the different colors indicate the different groupings. The nodes of the different colors represent the microbial groups. These play an important role in the group, as represented by the color: one color circle represents a biomarker, and the upper right corner is the biomarker name. The yellow node table shows the microbial groups that do not play an important role in the different groups. **(B)** *Proteobacteria, Neisseriaceae*, and *Staphylococcaceae* presented with significant changes in women who underwent cesarean section, with an LDA score ranking within the top 3.

The investigators further applied LEfSe and LDA to estimate the influence of CS on flora abundance. The results revealed that there was no significant difference in the bacterial flora between the CS endometrium and control endometrium, while the cervix *Proteobacteria, Neisseriaceae*, and *Staphylococcaceae* exhibited significant changes in CS women, with an LDA score ranking at the top 3 (Figures 5A,B).

**Figure 6:**
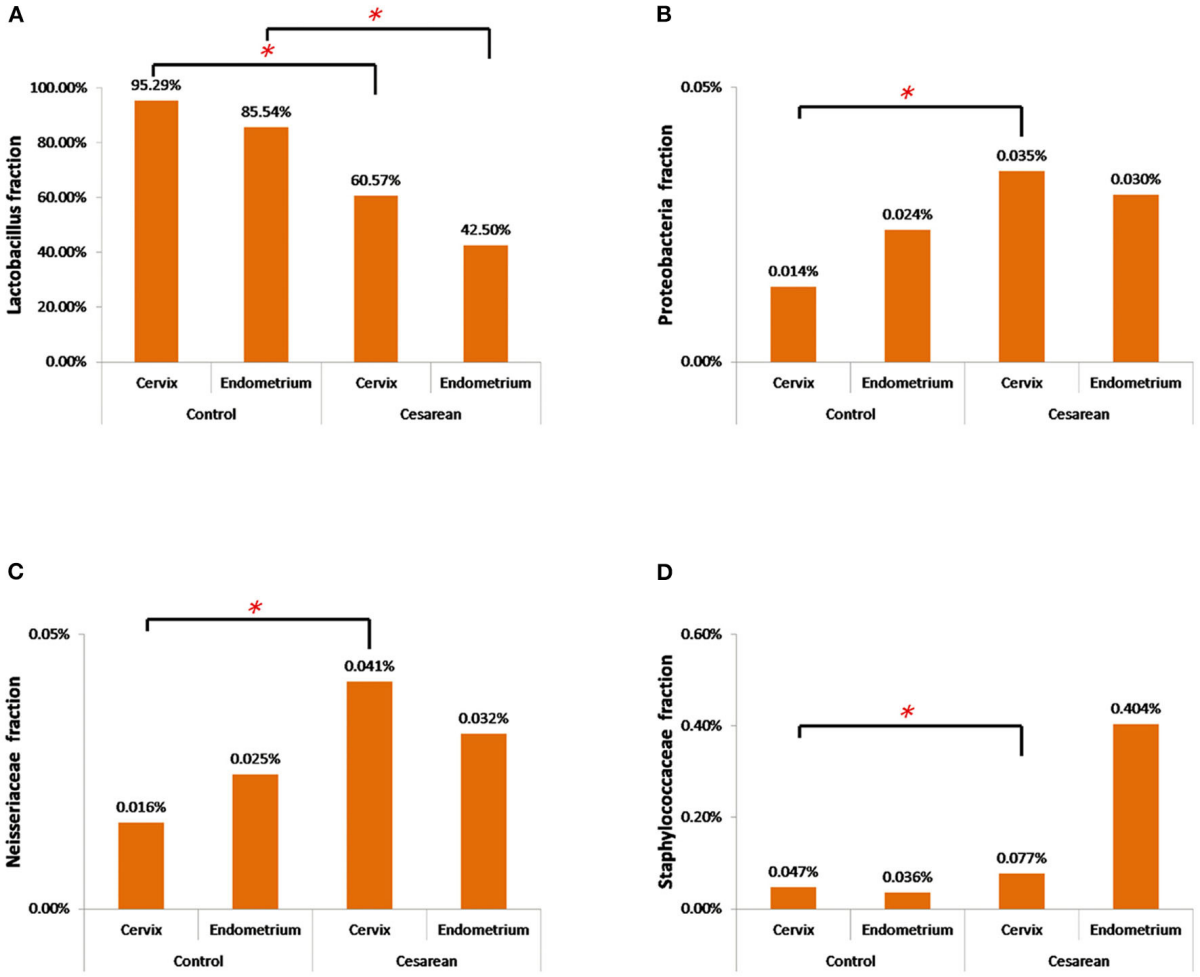
The abundance of the four significantly changed bacterial flora. **(A)** The abundance of *Lactobacillus* in the cervix and endometrium in the control group was significantly higher than that in the cesarean section group. **(B)** *Proteobacteria* had the highest abundance in the cesarean section group, especially in the cervical tissue. **(C)** *Neisseriaceae* had the highest abundance in the cesarean section group, especially in the cervical tissue. **(D)** *Staphylococcaceae* only increased in the cesarean section endometrium group. *P < 0.05.

The further analysis revealed that the abundance of *Lactobacillus* in the cervix of the control group was significantly higher when compared with the CS groups (Wilcoxon rank-sum test, *P* = 0.038). The highest abundance of *Proteobacteria* and *Neisseriaceae* was in the CS groups, especially in the cervical tissues (Wilcoxon rank-sum test, *P* = 0.001 and *P* = 0.025), while *Staphylococcaceae* only increased in the CS endometrium group (Wilcoxon rank-sum test, *P* = 0.038; Figure 6).

### The Correlation Between *Staphylococcaceae* and Cytokines

**Figure 7:**
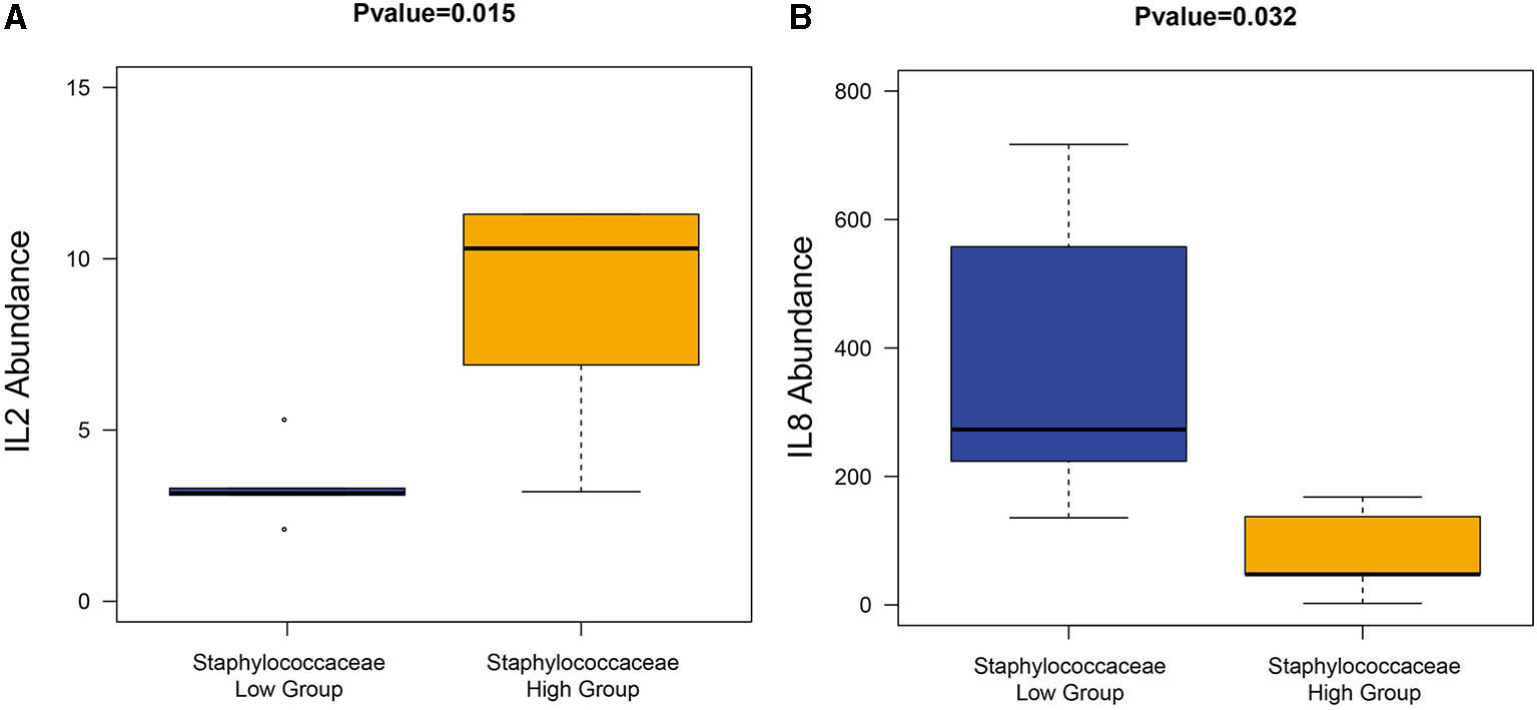
The correlation between *Staphylococcaceae* and cytokines. The high *Staphylococcaceae* abundance group sample had a significantly high abundance of IL2 **(A)** and IL8 **(B)**.

Since *Staphylococcaceae* is the genus that most dramatically changed in the CS endometrium group, the endometrium samples were re-divided into two groups according to the median abundance of *Staphylococcaceae* (the cut-off level was 2%). The high abundance of *Staphylococcus* was correlated to the significantly higher amount of IL-2 and lower amount of IL-8 (Wilcoxon rank-sum test, *P* < 0.05; Figure 7).

## Discussion

The potential mechanism of decreased fecundity for women with PCSD has not been fully elucidated. In the present study, 16S rDNA sequencing analysis was used to profile the characteristics of the microbial flora in the paired cervix and endometrial samples of CS and control women. Compared with the control women, the uterus *Lactobacillus* abundance in the cervix of CS women significantly decreased, and this was accompanied by the increase in the abundance of *Proteobacteria, Neisseriaceae*, and *Staphylococcaceae*. It is particularly noteworthy that the increased abundance of *Staphylococcaceae* in the CS endometrial tissues had a positive correlation with the IL2 amount and a negative correlation with the IL-8 amount.

A previous comparative study revealed that the percentage of the endometrial Lactobacillus in the healthy volunteers was highly stable. For the patients who became pregnant after the embryo transfer, the median percentage of uterus *Lactobacillus* was 96.45 ± 33.61%. The infertile women were shown to have a non-*Lactobacillus*-dominated (NLD) endometrium microbiota pattern. This was associated with infertility-related outcomes, such as reduced pregnancy rate, and the continued pregnancy rate even reduced the live birth rates (11). The data obtained from domestic animals also revealed a significant difference in the endometrium microbial community in sows with endometritis (12). In the present study, the interrupted microbial flora at each level in the infertile CS women had a similar pattern, and this also had a lesser *Lactobacillus*-dominating percentage in the CS groups. This change could be a sign of chronic endometritis and further impaired fecundity in women with uterine incision diverticulum.

Successful implantation is a highly coordinated process that involves changes in cytokines, adhesion molecules, hormones, enzymes, and growth factors (13). The cytokines at the maternal–fetal interface are essential for embryonic implantation and possess three main functions. First, these control the orientation of the embryo (5). Second, these regulate leukocyte trafficking at the implantation site to maintain the delicate balance between protecting the developing embryo and tolerating its Hemi-allogeneic tissues (14, 15). Third, chemokines participate in the processes of placental development and the promotion of human trophoblast migration (14). The T helper cells in the endometrium can be further subdivided into T helper 1 cells (Th1) and T helper 2 cells (Th2), depending on the pattern of cytokine production (16). Wegmann et al. were the first to develop the concept that during pregnancy, there is a shift from Th1 response to Th2, which functionally induces maternal tolerance and suppression (17). Consistent with this notion, the administration of Th1 interleukins IL-2 leads to fetal loss and preterm labor in mice (18). IL-8 is produced by Th2, epithelial cells, endothelial cells, and even human embryos (19). IL-8 is localized at the luminal, glandular epithelium, and endothelial cells throughout the menstrual cycle, with its expression being significantly higher during the peri-implantation periods (15). This has an important role in maintaining pregnancy due to its dual functions: the induction of chemotaxis in neutrophils and granulocytes, and the participation in innate immune response (20). The presence of IL-8 in the embryo culture medium is correlated to good clinical outcomes in *in vitro* fertilization [IVF; (19)]. In the present study, the correlation between *Staphylococcaceae* and cytokines reminded the possibility that disrupted microbial flora triggers specific signaling pathways at the maternal-fetal interface and leading to the production of pro- and anti-inflammatory cytokines, which further impaired fecundity in the CS group. A significant increase in the IL2 amount and decrease in the IL-8 amount in the high *Staphylococcaceae* group prompts that the mechanism of impaired fecundity in the CS group involves abnormal inflammation. The present data demonstrate the close connection of disrupted microbial flora with the abnormal local inflammatory reaction at the maternal-fetal interface, which further interfered with the embryo implantation procedure and decreased implantation. This potentially impaired fecundity increased the preterm labor and pregnancy loss/miscarriage rate reported in CS women (2). But the research has its limitation as a case-control study with a limited number of patients and a single menstrual period—follicular phase, so the results would be further certified with a Metagenome with a larger sample size.

In conclusion, the present study revealed the different composition of microbiota in the female reproductive anatomical locations (cervix and endometrium) between women with PCSD and women with previous vaginal delivery. The diverse inflammation cytokines abundance in the endometrial cavities were also analyzed. The dysbiosis condition in the CS uterus is closely correlated with the abnormal maternal immune system reaction at the maternal-fetal interface. These changes are further linked to the spectrum of clinical disorders related to the female reproductive system. Based on the data presented in the present study, further research on chemokines may assist in the endometritis diagnosis, and a therapeutic opportunity with *Lactobacillus* might be proposed. However, further research is still required to explore the mechanism of the complex host-microbiota interaction in the uterus in the different phases of menstruation and the confounding effect of a multitude of constitutional and environmental factors.

## Data Availability Statement

The datasets presented in this study can be found in online repositories. The names of the repository/repositories and accession number(s) can be found at: NCBI BioProject (accession: PRJNA753240).

## Ethics Statement

The studies involving human participants were reviewed and approved by the Ethics Committee of the Sixth Affiliated Hospital of Sun Yat-sen University. The patients/participants provided their written informed consent to participate in this study.

## Author Contributions

XY and GL have conceived and designed the study. XP made substantial contributions to the data analysis and interpretation. MC and BZ completed the data collection. XL and GL reviewed the content and critically revised it. All authors contributed to writing the manuscript.

## Funding

This research was funded by the following grants: Natural Science Foundation of China (Nos. 81671834 and 81971759), Special Support Program of Guangdong Province, Science and Technology Innovation Youth Talents Project (2016TQ03R444), and Pearl River S&T Nova Program of Guangzhou (201806010089).

## Conflict of Interest

The authors declare that the research was conducted in the absence of any commercial or financial relationships that could be construed as a potential conflict of interest. The reviewer YL declared a shared affiliation with the authors to the handling editor at time of review.

## Publisher's Note

All claims expressed in this article are solely those of the authors and do not necessarily represent those of their affiliated organizations, or those of the publisher, the editors and the reviewers. Any product that may be evaluated in this article, or claim that may be made by its manufacturer, is not guaranteed or endorsed by the publisher.
