# Supplementary figures and images for: Microbial Flora Changes in Cesarean Section Uterus and Its Possible Correlation With Inflammation
Source: Front Med (Lausanne). 2021 Nov 22;8:651938. doi: 10.3389/fmed.2021.651938 (PMC8645650; doi:10.3389/fmed.2021.651938)

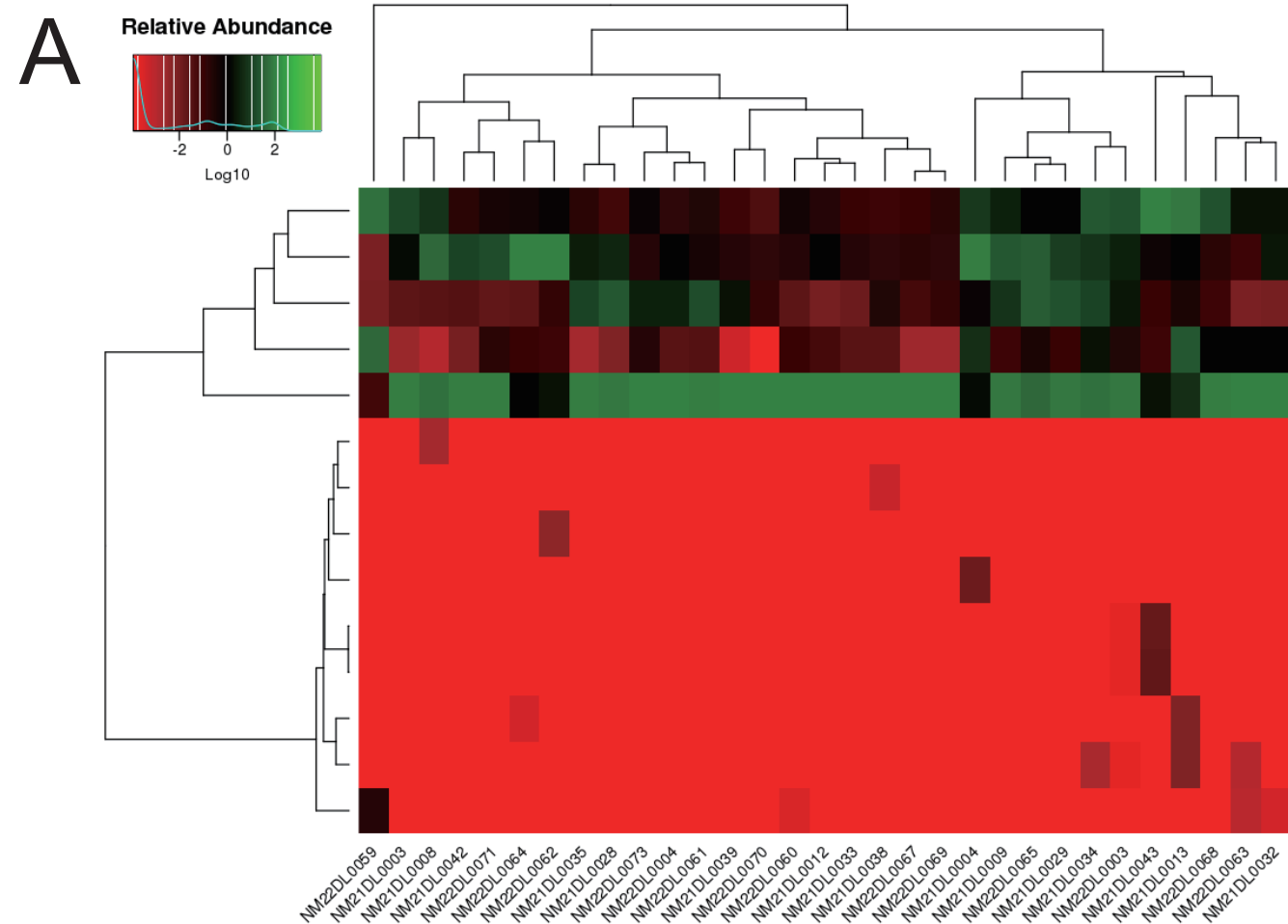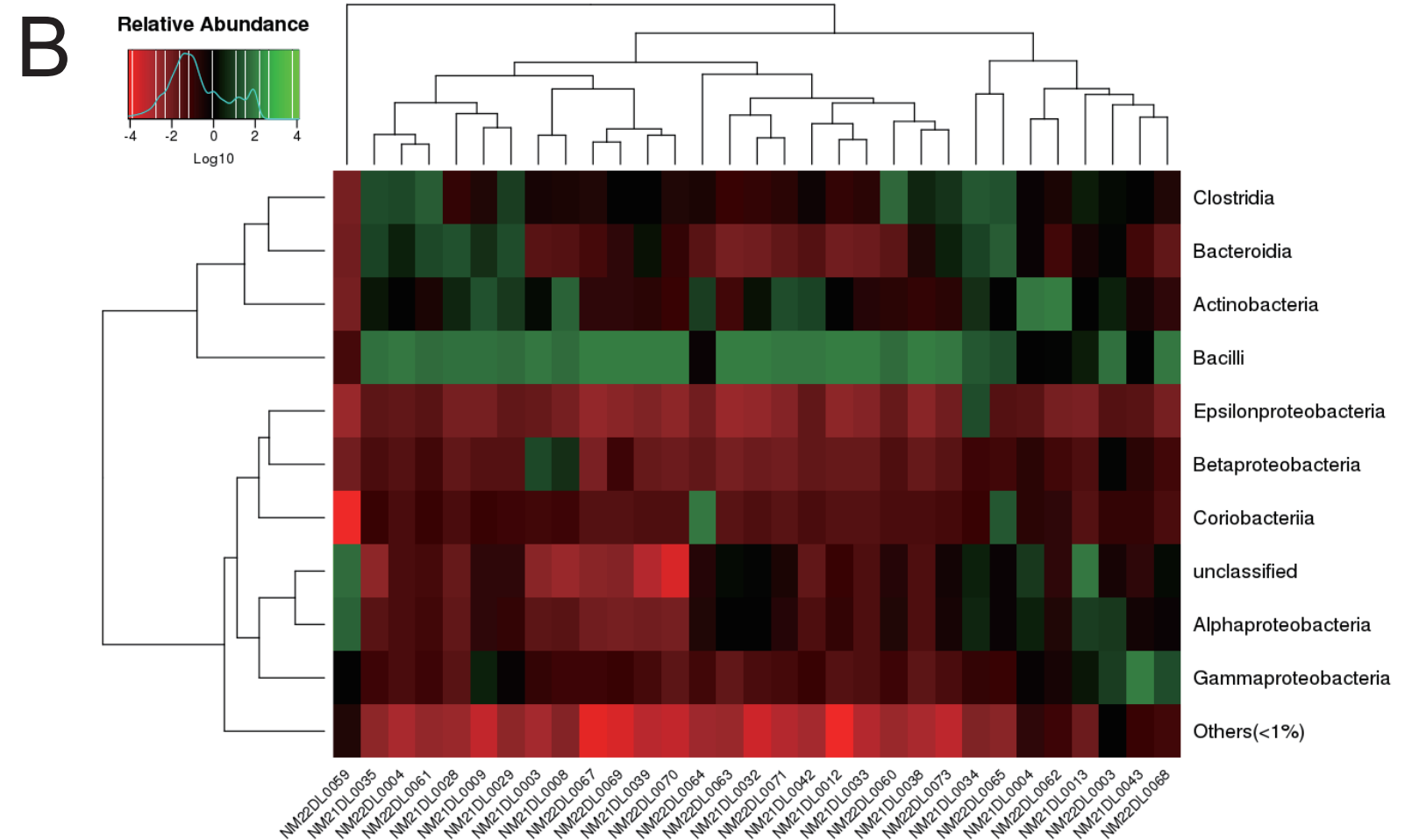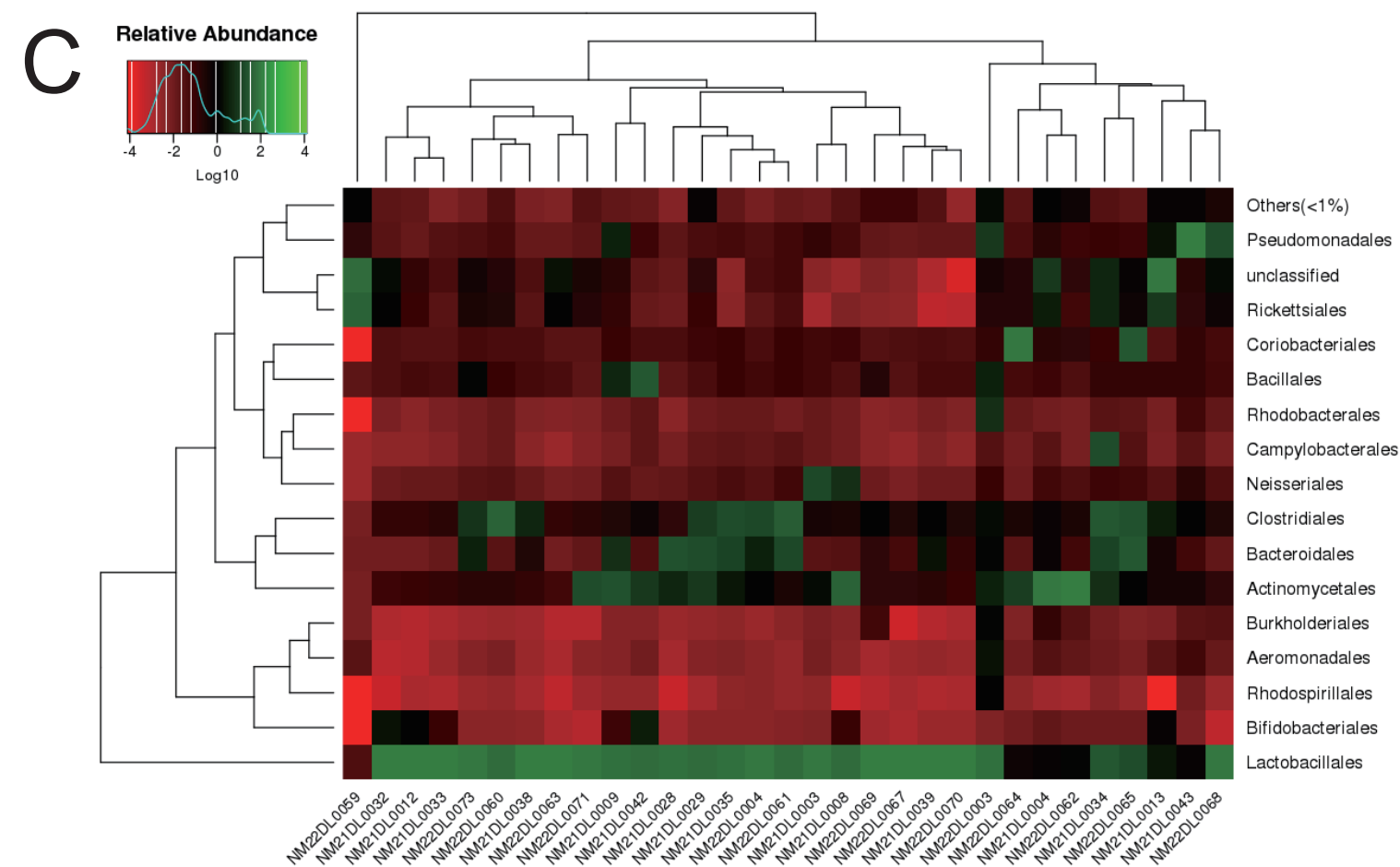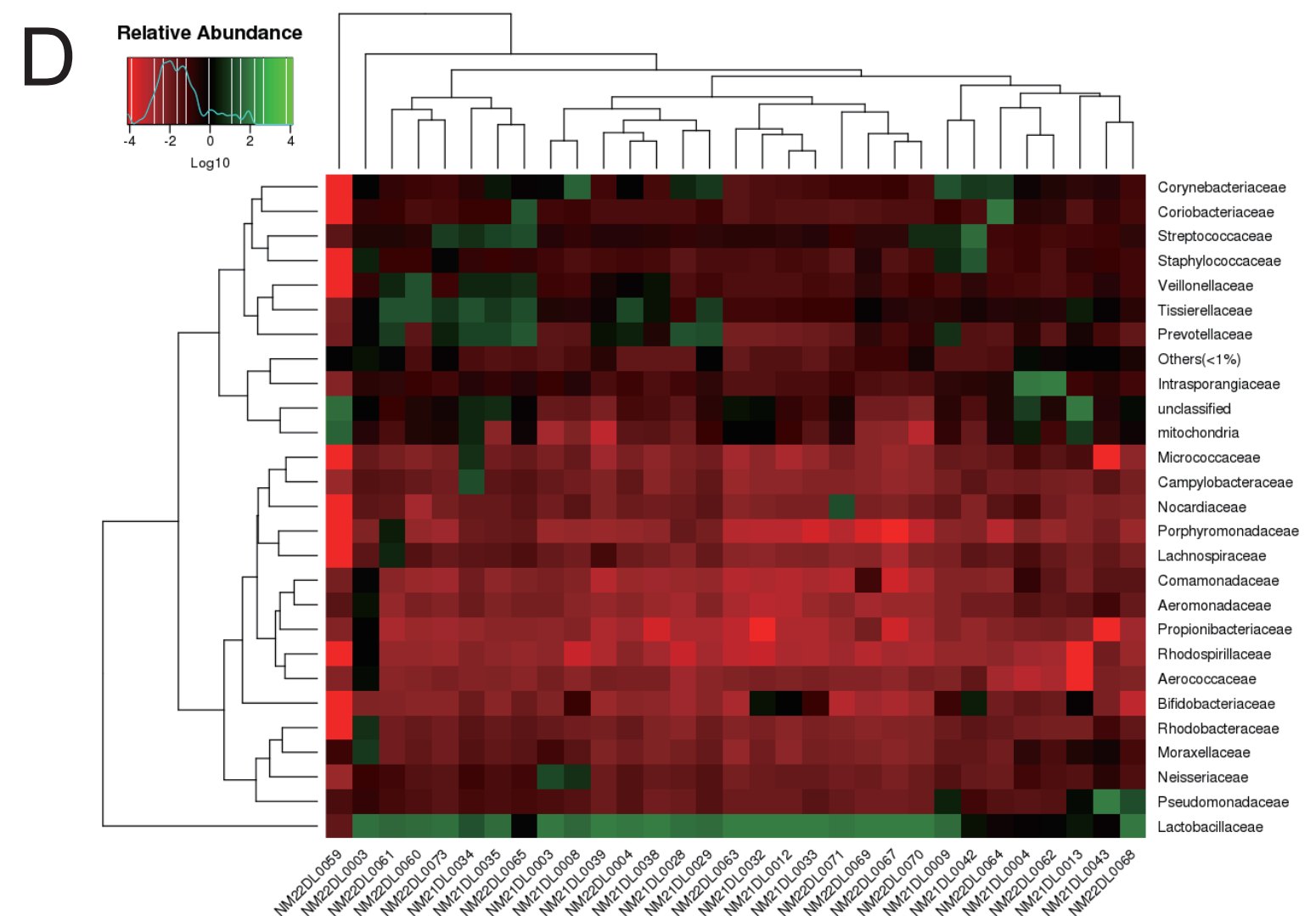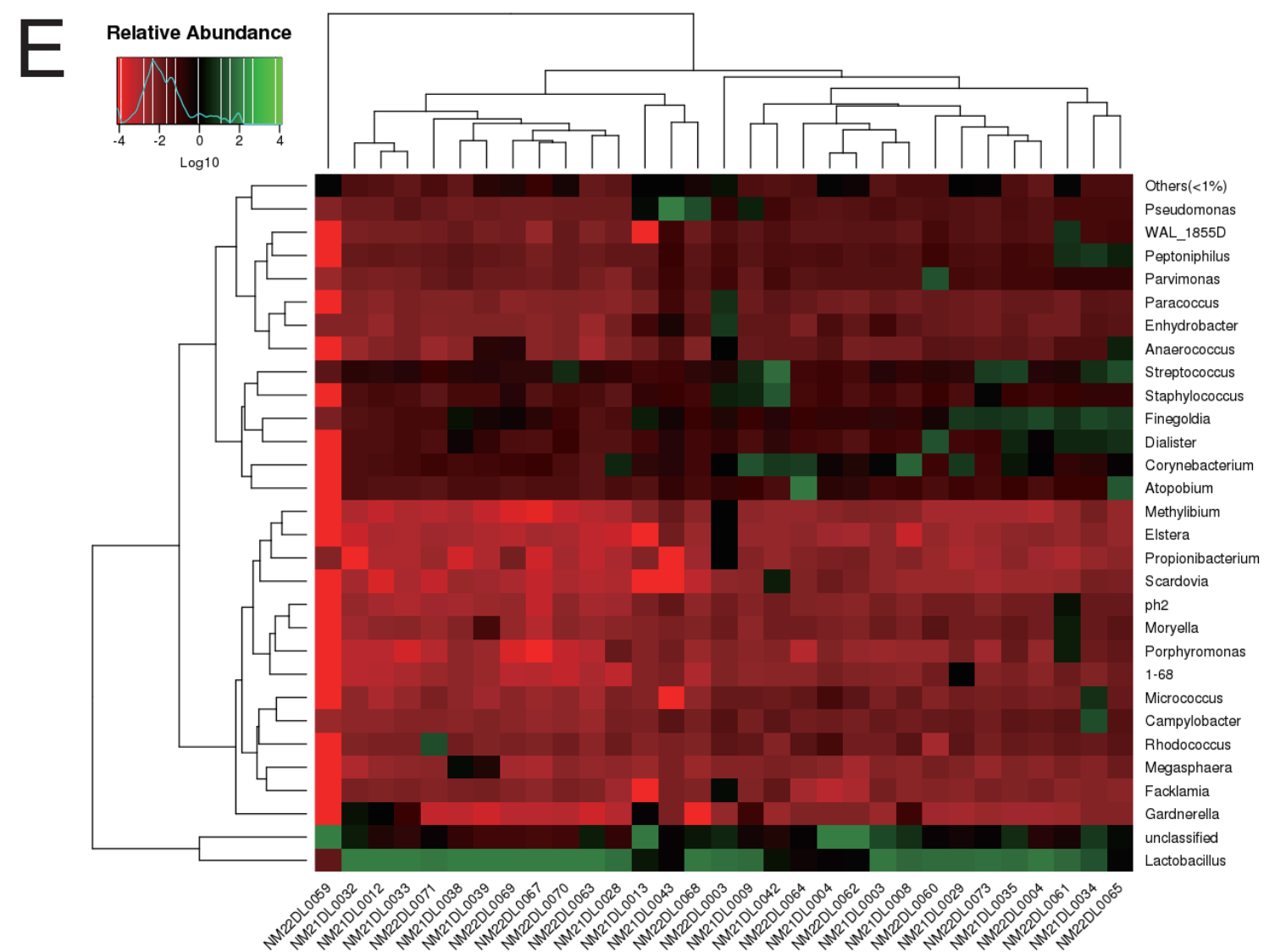

Supplement: Supplementary file 1 [file Image_1.pdf]
