# Supplementary material for: Microbial Flora Changes in Cesarean Section Uterus and Its Possible Correlation With Inflammation
Source: Front Med (Lausanne). 2021 Nov 22;8:651938. doi: 10.3389/fmed.2021.651938 (PMC8645650; doi:10.3389/fmed.2021.651938)
Supplement: Supplementary file 2 [file Table_1.DOCX]

The description of the supplement figure is as follows:(A) Heatmap at the phylum level; (B) Heatmap at the class level; (C) Heatmap at the order level; (D) Heatmap at the family level; (E) Heatmap at the genus level. The horizontal axis represent each species, and vertical axis represent each samples. The color scale bar ranged from -2.0 to 2.0, with green, black and red representing the low (green), medium (black), and high (red) microbial abundance, respectively.
